# Hyaluronic Acid Matrices for In Situ Measurement of Protein Diffusion Coefficients

**DOI:** 10.1002/elsc.70048

**Published:** 2025-10-07

**Authors:** Antonio C. F. dos Santos, Riya Debbarma, Kayla Hinton, Mazin Hakim, Ronghua (Andy) Bei, Luis Solorio, Eduardo Ximenes, Shiven Kapur, Vince Corvari, Michael Ladisch

**Affiliations:** ^1^ Laboratory of Renewable Resources Engineering Purdue University West Lafayette Indiana USA; ^2^ Department of Agricultural and Biological Engineering Purdue University West Lafayette Indiana USA; ^3^ Department of Engineering Boston College, Morrissey College of Arts and Sciences Chestnut Hill Massachusetts USA; ^4^ Weldon School of Biomedical Engineering Purdue University West Lafayette Indiana USA; ^5^ Eli Lilly and Company Indianapolis Indiana USA; ^6^ Department of Environmental and Occupational Health Indiana University Bloomington Indiana USA

**Keywords:** diffusion coefficients, hyaluronic acid, injectable biologics, monoclonal antibody, subcutaneous

## Abstract

In vitro measurement of protein diffusion within matrices that simulate the subcutaneous (SQ) environment is of interest, given that protein‐based therapeutics formulated for SQ injection comprise the largest class of biologics. To mimic the in vivo transport of a biologic from the SQ injection site through the extracellular matrix (ECM), in vitro diffusion assays typically utilize hyaluronic acid (HA) matrices, as it is the principal component of ECM. However, broad utility has been hampered by inherent lot‐to‐lot variability in commercially sourced HA, wherein key properties that impact protein diffusion (for example, molecular weight distribution and viscosity) differ across lots, even when nominal molecular weights are identical, making it challenging to compare results across matrices prepared from different HA lots. To address this gap, we report a facile approach wherein binary HA blends generated from individual HA matrices derived from distinct HA lots are functionally equivalent with respect to protein diffusion, that is, the diffusion of a representative set of proteins matches that in a previously reported single HA lot‐derived matrix that served as a representative reference. Taken altogether, our protocols enable preparing blended HA matrices with consistent diffusion properties, enabling the use of in vitro assays that leverage this capability.

*Practical application:* The measurement of in vitro diffusion of IgG‐type proteins enables calculation of diffusion coefficients that could help to guide the formulation of protein‐based therapeutics, administered by subcutaneous (SQ) injection, and used for treating a range of diseases, including cancer. The side‐by‐side comparison of these proteins over a period of time provides confirmation of consistency of properties when in vitro hyaluronic acid matrices, within which injected protein diffusion is measured, are also consistent. However, their broad utility has been hindered by the inherent variability of commercial sources of HA used to make‐up matrices that simulate the SQ environment in a predictable manner. Our research addresses this gap by defining an approach (validated with rheological and diffusion measurements) that facilitates the preparation of blended matrices from different lots of HA. The resulting matrix properties enable reliable measurement of protein diffusion from one lot to the next.

**Abbreviations**
ECM: extracellular matrix
HA: hyaluronic acid
ISM: in vitro Subcutaneous Matrix
SEC: size exclusion chromatography
SQ: subcutaneous

## 1 Introduction

Monoclonal antibodies (mAbs) constitute two‐thirds of current biopharmaceutical protein products with nearly 1200 antibodies at various stages of testing, worldwide, as of November 2022 [1]. In the United States, there are 122 mAbs approved by the Food and Drug Administration (FDA) as of June 2022 [2]. Among the approvals between 2014 and 2019, half were for injectable SQ formulations [3], partly because administration by automated SQ injection is preferred over more arduous and time‐consuming intravenous infusion methods [4, 5].

The need for high concentration, injectable biologic formulations for SQ delivery has motivated the development of pre‐clinical in vitro measurements that relate injectable biologic characteristics to their in vivo pharmacokinetic outcomes [6, 7, 8]. In developing in vitro assays simulating the SQ environment for evaluating biopharmaceutical protein products, hyaluronic acid (HA) and collagen appear as suitable materials for the artificial SQ matrix, given that they are the major structural components in the extracellular matrix of the SQ environment [9]. Indeed, artificial matrices made with HA can recreate key characteristics of the SQ environment, such as the negative charge, volume exclusion, hydrophilic character, and elasticity [10, 11].

A challenge, however, with using HA as the matrix for the in vitro assays is its inherent lot‐to‐lot variability since all commercial sources purify HA from biological source(s). Its properties, including molecular weight distribution and solution viscosity, can differ even when the nominal molecular weights of two different HA lots are the same. This, in turn, creates a significant barrier to the widespread use and adoption of these assays, given the inherent comparability issues that result from preparing SQ mimicking HA matrices derived from different HA lots. This challenge prompted us to develop and validate a generally applicable protocol for creating internally consistent HA matrices by mixing HA matrices from two given HA lots, regardless of their exact individual molecular properties. We further delineate criteria for determining when such mixing is successful. As a representative example of the applicability of our approach, we report a binary blend prepared from parent HA lots of average molecular weights (M_w_) 0.83 MDa and 1.64 MDa (as per the certificate of analysis provided by Lifecore Biomedical) to create a new matrix whose properties are equivalent to the properties of a reference matrix from a single‐source HA lot of 1.32 MDa.

We validated the protocols in a stepwise manner from two different but complementary perspectives. First, we characterized the molecular weight distribution and viscosity of binary blends of matrices and compared them with the properties of unmixed, reference matrices. We successfully showed that they matched. Second, we applied the protocols to a recently developed in vitro assay for measuring protein diffusivity in HA matrices, called the in vitro subcutaneous matrix (ISM) system [12]. We demonstrated that diffusion coefficients of the same proteins (i.e., bovine serum albumin (BSA), bovine‐IgG, and a mAb) stayed relatively unchanged when the reference matrix was replaced with a binary matrix blend prepared using the protocol reported in this manuscript.

Taken together, this study reports the formulation research, matrix testing, and acceptance criteria for preparing internally consistent HA matrices. Since the reference matrix will eventually be depleted and replaced by a new lot of material, a protocol that ensures equivalent properties and performance with respect to their use in determining protein diffusion coefficients, and potentially other applications, is required. This approach paves the way for broader adoption of HA‐based in vitro assays by ensuring consistency across HA lots.

## 2 Materials and Methods

Different HA lots and sizes (Table 1) sourced from Lifecore Biomedical (Chaska, MN) (Table 1) were formulated (Table 2) for evaluating the impact of matrix composition on diffusion coefficients at different initial protein concentrations for BSA, b‐IgG and a monoclonal antibody (mAb3) where the three proteins had different MW, pI, and charge at pH 7.4 (Table 3). The results from the assays reported here were used to define acceptance criteria for the HA matrices used in the in vitro Subcutaneous Matrix (ISM) system and compare diffusion characteristics of the mAb to those of BSA and b‐IgG.

**TABLE 1:** Sodium hyaluronate powder characteristics by lot as purchased.

Matrix ID	Lot #	Nominal molecular weight (MDa)^a^	Average MW (MDa)^a^	Catalog (Lifecore)
A	028475	1.5	1.32	HA15M‐5
B	025735	1.5	1.67	HA15M‐5
C	028605	1.5	1.64	HA15M‐5
D	27409	1.0	0.83	HA1M‐5
E	24051	0.7	0.69	HA700K‐5
F	29817	0.5	0.381	HA500K‐5

^a^ Data from the certificate of analysis as provided by Lifecore Biomedical. Average MW measured indirectly by viscosity measurement.

**TABLE 2:** Composition and properties of hyaluronic acid matrices used in this work.

Matrix ID	HA (size MDa^a^)		HA concentration/Blend composition (mg/mL)	Viscosity (Pa.s) at 0.02 Hz	M_n_	M_w_	Polydispersity
***A***	***1.32***		***10***	***7.2*** + ***0.1***	***1.15*** + ***0.04***	***1.58*** + ***0.02***	***1.37***
B	1.67		10	14.4 + 0.3	1.23 + 0.05	1.65 + 0.03	1.34
C	1.64		10	17.12 + 0.01	0.96 + 0.09	1.40 + 0.04	1.46
D	0.83		10	1.01 + 0.01	0.84 + 0.04	1.30 + 0.03	1.54
E	0.69		10	0.55 + 0.02	0.63 + 0.03	1.06 + 0.01	1.70
F	0.381		10	0.20 + 0.09	0.44 + 0.04	0.67 + 0.07	1.54
BD	1.67	0.83	B (6) + D (4)	8.5 + 0.1	1.18 + 0.09	1.69 + 0.06	1.44
*CD*	*1.64*	*0.83*	*C (6)* + *D (4)*	*7.4* + *0.5*	*0.96* + *0.06*	*1.59* + *0.02*	*1.66*
BE	1.67	0.69	B (7) + E (3)	8.2 + 0.3	1.26 + 0.08	1.79 + 0.04	1.42
CE	1.64	0.69	C (7) + E (3)	4.8 + 0.2	1.11 + 0.06	1.64 + 0.03	1.48
*BF*	*1.67*	*0.381*	*B (7.5)* + *F (2.5)*	*7.6* + *0.6*	*0.99* + *0.07*	*1.69* + *0.02*	*1.71*
CF	1.64	0.381	C (7.5) + F (2.5)	8.0 + 0.6	1.11 + 0.08	1.70 + 0.04	1.54

^a^ Data from the certificate of analysis as provided by Lifecore Biomedical. Average MW determined indirectly by viscosity measurement with reference HA matrix, denoted in bold italics. Mixtures selected for runs with mAb3 are indicated in italics. M_n_ and M_w_ were obtained from size exclusion chromatography (SEC).

**TABLE 3:** Properties of proteins examined in this work.

Protein	Molecular weight (kDa)	pI	Extinction coefficient (10 mg/mL, 1 cm path)	Zeta potential in PBS pH 7.4 (mV)	Hydrodynamic radius of protein (nm)
BSA	67	4.7	6.7	−8.6 + 0.1	3.2 + 0.2
Bovine IgG	150	7	13.7	−2.4 + 0.1	5.3 + 0.2
mAb3	150	6.2	16.3	−6.6 + 0.7	4.4 + 0.2

### 2.1 Experimental Methods and Diffusion Calculation

The ISM method for measuring unlabeled proteins within a hyaluronic acid gel over time has been previously described with changes in experimental conditions for this work as noted below [12]. A repurposed cell culture chamber was filled with 6.6 mL HA (6 mm depth), and unlabeled proteins were detected within the HA gel using their intrinsic fluorescence (trp‐based). Previously, samples were excited with UV‐B light (280 nm optimum absorbance) and their fluorescence imaged at 384 nm using the Gel Doc EZ Gel Documentation System (BioRad).

The method was further developed with improved sensitivity using a higher resolution Gel Doc Imaging System (BioRad 6.3 MP CMOS detector and pixel size 2.4 × 2.4 µm), where the sample is excited at 302 nm and imaged at 590 nm.

Images were captured every 30 min for 4 h. The images were then converted to concentration maps. Concentration‐dependent diffusion was calculated using equation (1) δC/δt = D ∇^2^C + δD/δC(δD/δx)^2^ + δD/δC(δD/δy)^2^ [13]. The resulting function was summarized into a single value by calculating the average diffusion and the median protein concentration above the minimum detection protein concentration (2.5 mg/mL) [14]. Two replicates with 3 injections each were done for each experiment. Injection volume was 20 µL for protein concentrations of 100 mg/mL and 10 µL for initial protein concentrations > 100 mg/mL. The different volumes were used to reduce overlap between the outer edges of triplicate injections, which occur at the higher concentrations. The impacts of different injection volumes are discussed below.

### 2.2 Hyaluronic Acid Matrix Preparation

Sodium hyaluronate powder was purchased from Lifecore Biomedical (Chaska, MN). Table 1 describes the different lots, with their associated nominal, actual, and measured molecular weights. 0.01 M Phosphate‐buffered saline (PBS) (containing 0.138 M NaCl and 0.0027 M KCl, pH 7.4) was prepared by combining 5 L pre‐weighed mix (Sigma Aldrich, St. Louis, MO) with autoclaved deionized water (DI H_2_O). While the manufacturer provided a certificate of analysis for different lots of HA in the same MW range were similar, there were lot‐to‐lot differences in the MW of the polymers that resulted in matrices with different viscosities, hindering the consistent determination of protein diffusion at equivalent protein concentrations, as diffusion is a function of viscosity.

Matrices based on individual HA lots were prepared by addition of 200 mg 1.5 MDa HA to 40 mL PBS (pH 7.4) in a borosilicate glass, round bottom centrifuge tube (DWK Life Sciences, Millville, NJ, USA), capping the tube and mixing for 2–4 h at low speed using a Rotisserie Tube Rotator (Scilogex, Rocky Hill, CT, USA) at room temperature. Addition of a second aliquot of 200 mg 1.5 MDa HA was followed by mixing overnight and then transferred to a refrigerator (4°C), where the formed gel was degassed for an additional 24 h, resulting in a clear viscous matrix that may be stored at 4°C for up to 2 weeks before use [12].

For matrix blends containing a combination of two HA lots, where each lot consisted of a different HA molecular weight fraction, the method above was adapted so that the HA was added to the buffer in three aliquots with a minimum of 90 min mixing between the additions. The first two aliquots were the larger molecular weight polymer, and the third was the smaller polymer. Table 2 summarizes the matrix identifiers and compositions of the different single‐component (A to F) and binary (BD to CF) matrices.

The polymer molecular weights were also measured using size exclusion chromatography (SEC) carried out at room temperature (20°C). The different matrix samples were first diluted 10x in PBS in order to achieve a concentration within the working range of the SEC column. Prior to SEC analysis, each sample was syringe‐filtered through a 0.2 µm filter. A Waters e2695 Separations Module (Waters Corporation, Milford, MA, USA) was used to store samples at 4°C before injection. The injection volume was 20 µL with PBS buffer as the mobile phase at a 1 mL/min flow rate. Two columns in series—PL aquagel‐OH 60 (7.5 × 300 mm, 8 µm) and PL aquagel‐OH 40 (7.5×300 mm, 8 µm), both acquired from Agilent Technologies (Santa Clara, CA, USA)—preceded by a guard column, were used for SEC at room temperature. Polymers were detected using two inline detectors, that is, 2414 Refractive Index detector and 2489 UV detector (220 nm) (Waters Corporation, Milford, MA, USA). Data were collected for 30 min. Molecular weight was determined using Empower 4 (Waters Corporation, Milford, MA, USA), and the system was calibrated using InfinityLab EasiVial PEO/PEG Standards (Agilent Technologies, Santa Clara, CA, USA) [15]. The matrices, once formulated and characterized, were placed into the ISM devices as described previously [12].

### 2.3 Rheological Characterization

The rheology of each HA matrix was characterized using an AR 2000 rheometer (TA Instruments, New Castle, DE, USA). Each test was done at 25°C using a 40 mm, 2‐degree cone with a truncation gap of 52 µm. A volume of 590 µL of each sample was used per replicate. The system was allowed to equilibrate and reach the testing temperature for 2 min. A shear sweep from 0.01 to 100 Hz was performed for 60 s or until steady state was reached. All samples were measured in triplicate.

### 2.4 Proteins

Protein characteristics are summarized in Table 3. Internal standards and control proteins were bovine serum albumin (BSA), lyophilized powder (lot# SLCC9421, Sigma‐Aldrich, St. Louis, MO, USA), and IgG from bovine serum (b‐IgG), lyophilized powder (lot# SLBZ8713, Sigma‐Aldrich, St. Louis, MO, USA). To the 5 L PBS buffer, 2.5 mL of 0.05% Tween 80 (Sigma‐Aldrich, St. Louis, MO, USA) was added, and weighed amounts of proteins were dissolved in the PBS + Tween 80 buffer. Concentrations were determined by extinction coefficients that inherently correct for % moisture. The proteins were dissolved in 20 mL PBS + Tween 80 in two 1200 mg aliquots (2400 mg protein total), resulting in 120 mg/mL stock solutions in 50 mL, capped centrifuge tubes (Corning Inc., Corning, NY, USA). Dissolution was assisted by inverting the tubes at low speed in the Rotisserie Tube Rotator (Scilogex, Rocky Hill, CT, USA) overnight at room temperature. The tubes were then stored at 4°C for up to 2 weeks until use.

A monoclonal antibody (mAb3) was provided by Eli Lilly at 150 mg/mL. The solution was dialyzed using Slide‐A‐Lyzer G1 dialysis cassettes with 10 kilodaltons (kDa) molecular weight cutoff (Fisher Scientific, Pittsburgh, PA, USA). The final buffer was the same PBS + Tween 80 buffer described above. The buffer volume used was 1 L, replaced 7 times. Dialysis was performed at 4°C to avoid precipitation.

Protein stock solutions were diluted with PBS + 0.05% Tween 80 (v/v) to prepare internal standard solutions (14 or 28 mg/mL) or 25, 50, 75, 100, and 150 mg/mL samples for diffusion measurements. Concentrations (average of 4 replicates) were measured using 280 nm absorbance with Take 3 nanoplates in an Epoch 2 microplate spectrophotometer (BioTek U.S., Winooski, VT, USA). Protein size and zeta potential were measured in triplicate using a Zetasizer Nano ZS90 (Malvern Panalytical Inc., Westborough, MA, USA) at 1 mg/mL with 1 mL total volume in disposable cuvettes (Fisher Scientific, Pittsburgh, PA, USA) pipetted into a universal zeta potential dip cell (Malvern Panalytical Inc., Westborough, MA, USA). The protein standard was diluted with water since Tween 80 and high salt concentrations interfere with Zetasizer measurements by decreasing the overall surface charges and shifting the zeta‐potential of proteins toward zero [16]. Injection of protein into the HA matrix using an injection guide and ISM device was performed as described previously [12].

### 2.5 Acceptance Criteria for Specific Runs

Criteria noted below (i to iv) were applied after each run and used to determine whether a specific experiment(s) should be repeated. Fluorescence (pixel intensity) was analyzed and compared against the internal standard—b‐IgG at 14 and 28 mg/mL initial protein concentrations—for a series of diffusion experiments carried out over a period of 4 months using freshly prepared HA matrices each time. The coefficient of variation was 8.2% for 14 mg/mL and 7.7% for 28 mg/mL. These values indicate minimal instrumental drift or variation over this time period. Previously developed protocols for the ISM system [13, 14] were applied to the analysis of the data to specify acceptance criteria for specific runs. These were:

- Protein solution concentration must be within 5% of the nominal value as measured by the extinction coefficient.
- HA background pixel intensity must be < 4000.
- Mass balance for protein must be > 80% and < 125% as calculated using the internal standard.
- The two replicates must be within 30% of each other.

A total of 24 experiments were carried out for this paper, and of these, 3 did not meet the acceptance criteria, resulting in an 87% success rate. These experiments were re‐run. All reruns met the acceptance criteria i—iii. The re‐runs that also met criteria iv replaced the first experiments that had not met the acceptance criteria. The runs were combined with the original experiments, and their results were reported together.

## 3 Results

### 3.1 Matrix Characterization and Reproducibility

Table 2 summarizes all the matrices that were prepared and analyzed. Matrix A (row 1, bold, Table 2), used in the previous work to develop the ISM system [12], was prepared with 10 mg/mL of HA lot #028475. Matrix A served as the reference standard for blends of matrices subsequently formulated with other lots of HA of different molecular weights (Table 2). The acceptance criterion for a new lot of matrix is based on achieving comparable diffusion coefficients for the same proteins that serve as an internal standard: that is, BSA and b‐IgG. We also sought to identify other characteristics that would indicate the suitability of a matrix and considered both SEC and viscosity. Rheology of the different matrices (A, CD, BF) showed the HA matrices to be shear thinning, which is a characteristic associated with the viscosity of the matrix and reflected by the difference in diffusion coefficients, as indicated later in the Section 3. Matrices having equivalent viscosities were found to be a good indicator of consistency, as discussed below.

**FIGURE 1:**
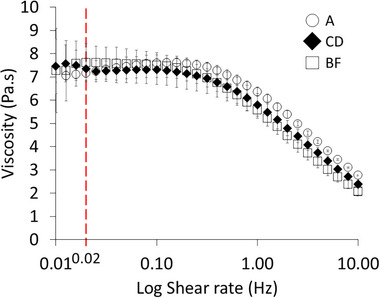
Apparent viscosity plotted against the shear rate for selected hyaluronic acid matrices. Error bars correspond to standard error of three replicates. Dashed bar corresponds to the 0.02 Hz shear rate, which is used to compare different matrices' viscosities. Matrix composition is described in Table 2.

**FIGURE 2:**
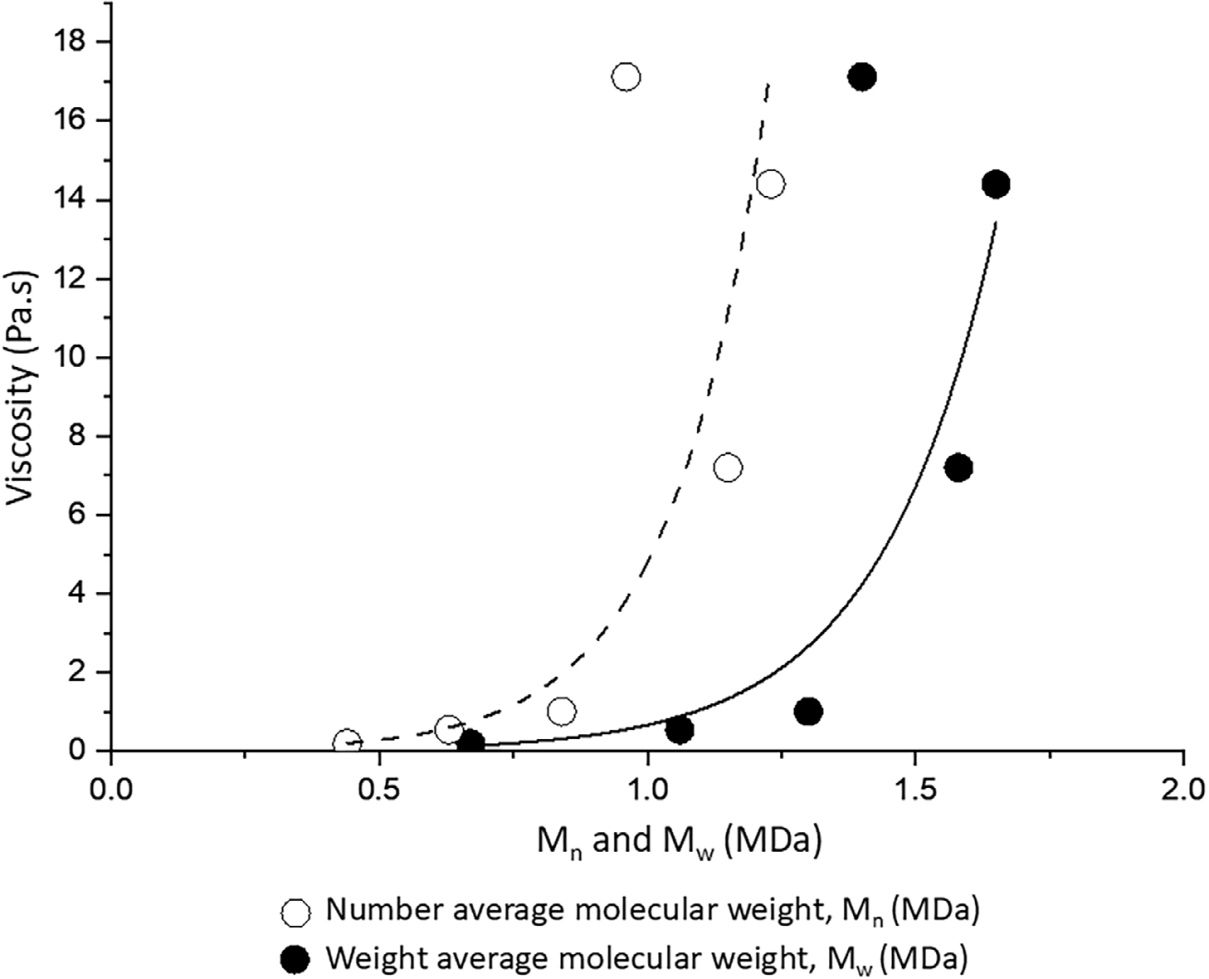
Apparent viscosity of different hyaluronic acid matrices. All solutions were made with a single HA lot at 10 mg/mL PBS. Reported value for apparent viscosity is the average of triplicate measurements.

The viscosity of HA at 10 mg/mL, measured at a shear rate of 0.02 Hz and plotted against the number average molecular weight (M_n_) and weight average molecular weight (M_w_) determined by SEC (Figure 2), showed that HA viscosity increases exponentially with M_n_ and M_w_:

(1) $$\mu =0.018\text{exp}(5.6xM_{n})$$

(2) $$\mu =0.007\text{exp}(4.6xM_{w})$$

with the fit of the data in Figure 1 corresponding to Equations (1) and (2). Hence, the blend compositions were calculated to match the total number of monomers present within the matrix (as indicated in the second column of Table 2), with the resulting matrices that most closely matched the viscosity of matrix A (7.2 ± 0.1 Pa·s) being BF (7.6 ± 0.6 Pa·s) and CD (7.4 ± 0.5 Pa ·s).

Blends BF and CD were then further compared based on diffusion coefficients determined for BSA and IgG using the ISM method 14, 15, 16 While BD, BE, and CF also had similar viscosities, their viscosities were higher than the reference HA, and hence these combinations were not considered further for this paper.

It should be noted that the protocol given here could also be used to formulate matrices designed to test different characteristics of HA, such as those found in a tumor environment [15] or for SQ environments found at different anatomic locations. SEC of the different matrix formulations (Table 2) shows that B and C have similar molecular weight distributions but distinct polydispersities (1.34 and 1.46, respectively). Since no external shear stress is applied to the HA matrix in the ISM device during the determination of diffusion coefficients of proteins at equivalent concentrations in these HA matrices, zero‐shear viscosity (corresponding to shear rate 0.01 – 0.1 Hz in Figure 1) is indicated to be a useful and readily measurable indicator for selecting equivalent matrices.

Blends containing HA fraction B had polydispersities of 1.44, 1.42, and 1.71 for BD, BE, and BF, respectively, compared to blends with C, where the polydispersities were 1.66, 1.48, and 1.54 for blends CD, CE, and CF, respectively. An increase in polydispersity was expected due to the blending of two HA lots with markedly different molecular weights.

The impact of M_n_ and the average molecular weight M_w_ were different for blends with B and C. The blends with B had statistically significant (*p* value < 0.05) changes, where M_n_ decreased from 1.23 ± 0.05 MDa to 0.99 ± 0.07 MDa for BF compared to B, where M_n_ decreased from 1.23 ± 0.05 MDa (for B) to 1.18 ± 0.09 MDa for BD. For the blends made with C, M_n_ values were not statistically different from the C matrix. In comparison, M_w_ for the C blends were statistically different, increasing from 1.40 ± 0.04 MDa for C to 1.59 ± 0.02, 1.64 ± 0.03, and 1.70 ± 0.04 MDa, for CD, CE, and CF, respectively. Moreover, it has to be noted that the individual HA lots are polydisperse, and the HA blends consist of two different HA molecular weights, with a higher proportion of the larger molecular weight HA. This skews the calculated M_n_ and M_w_ toward higher values (Table 2). Consequently, the zero‐shear viscosity measurements were considered to be more suitable for direct comparison and validation of matrix combinations since these incorporate interactions between HA polymers that result in viscosities that affect diffusion coefficients for the injected proteins.

### 3.2 Reproducibility of Protein Fluorescence and Diffusion Over Time

The reproducibility of diffusion measurements for BSA in reference matrix A was tested by two separate experiments carried out 160 days apart—with a total of 12 injections for each experiment (4 devices × 3 injections each). Three starting protein concentrations (25, 50, and 75 mg/mL) were used. The average diffusion coefficients and their associated coefficient of variation were 5.6 × 10^−7^ cm^2^/s and 5.4% at 25 mg protein/mL; 5.4 and 6.7%; and 5.2 and 14.0%, at 50 mg/mL and 75 mg/mL, respectively. This showed that the instruments and the method itself are reproducible.

The image preprocessing functions utilized for this manuscript were previously described and are based on the moving median of the pixel values in the area surrounding a given pixel [12]. Image preprocessing is effective in removing outliers and other artifacts. Since effective diffusion is a function of the measured concentration, removing outliers is required for accuracy and reproducibility. Conversely, if a small area of high protein concentration at the injection site resembles common outliers (dust and air bubbles) due to a value that is significantly higher than its surroundings, that area is also filtered through image processing, thus limiting the range of protein concentrations that can be presently analyzed. Further development of image processing is required to overcome this challenge.

### 3.3 Diffusion Measurements on Different HA Matrices

The effective diffusion was measured in the different matrix formulations (Table 2) at protein concentrations of 50 to 55 mg/mL (Table 4). Variations in median protein concentrations (right column in Table 4) reflect slight variations of the matrix within each ISM device. Differences in the diffusion coefficients for BSA and IgG reflect these concentrations as well as the known concentration‐dependent behavior of BSA and IgG, which was measured with Matrix A [14]. The diffusion coefficient for mAb3 was independent of concentration across different matrices, unlike b‐IgG and BSA, which exhibit concentration‐dependent behavior. The exact cause of the different behaviors is not known, although we postulate pH‐dependent protein aggregation as well as charge may play a role, as also suggested by data of [16, 17, 18].

**TABLE 4:** Calculated effective diffusion and corresponding concentrations for proteins at 50–55 mg/mL in different matrices.

Protein	Matrix	Effective diffusion × 10^−7^ (cm^2^/s)	Median protein concentration after 4‐h diffusion (mg/mL)
BSA	A	4.72 + 0.05	7.8 + 0.0
	B	4.60 + 0.04	17.4 + 2.7
	BF	4.72 + 0.31	9.5 + 0.1
	CD	5.19 + 0.08	4.8 + 0.8
b‐IgG	A	2.37 + 0.05	3.5 + 0.1
	B	1.44 + 0.30	16.7 + 0.1
	BF	0.76 + 0.02	9.82 + 1.5
	CD	0.88 + 0.18	12.8 + 5.8
mAb3	A	2.37 + 0.25	7.5 + 0.4
	B	2.24 + 0.12	12.3 + 0.0
	BF	2.90 + 0.06	8.3 + 0.5
	CD	2.52 + 0.10	16.0 + 1.6

### 3.4 Diffusion of mAb3 Measured at Different Protein Concentrations

**FIGURE 3:**
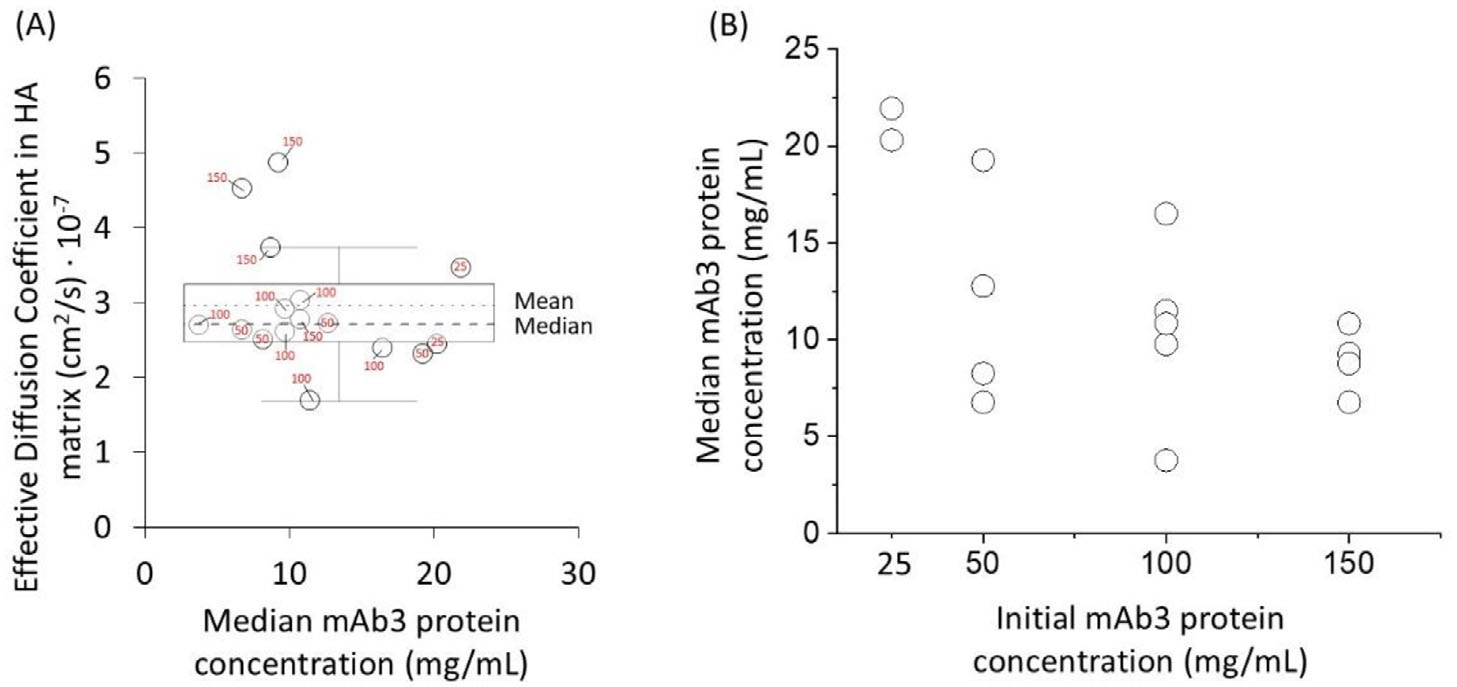
Calculated effective diffusion coefficient of mAb3 at different initial concentrations (25–150 mg/mL). Each point is an experimental replicate (3 injections) in matrices A and CD (Table 2). (a) Box plot of the effective diffusion coefficients versus the median protein concentration measured during the experiment at *t* = 60 min. The box corresponds to the interquartile range (IQR). The whiskers are the 95% confidence interval. Dotted line is the mean value, and the dashed line is the median value. The points outside the whiskers are outliers. The initial protein concentrations are shown in red font. (b) Plot of the median protein concentration versus the initial protein concentration indicates that the former is independent of the latter (*R* ^2^ = 0.32).

The possible concentration dependence of mAb3 diffusion within HA matrices was further tested over mAb3 concentrations varying from 25 to 150 mg/mL. Experiments used the reference Matrix A and the blended matrix CD. For Matrix A, the initial protein concentrations were 25, 50, and 100 mg/mL, and for blended matrix CD, 50, 100, and 150 mg/mL. Two replicates with 20 µL injections (6 total injections) were used in the Matrix A, and for 50 mg/mL in blended matrix CD. For 100 and 150 mg/mL mAb3 injected into the blended matrix CD, four replicates of 10 µL injections (12 total injections) were used. Results are summarized in Figure 3, with distribution of individual replicate measurements indicating that the effective diffusion coefficient was independent of the median protein concentration after 4 h and that the median protein concentration was independent of the initial protein concentration. Two outliers were identified out of the 16 experiments due to being outside the interquartile range for the complete dataset, and were removed from further analysis.

### 3.5 Reproducibility of Replicate mAb3 Diffusion Measurements With Blended Matrices Over Time

**FIGURE 4:**
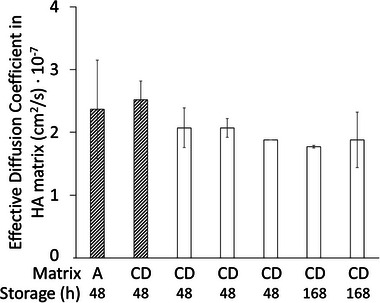
Calculated effective diffusion coefficient of mAb3 (50 mg/mL initial concentration) in matrices A and CD stored at 4°C with extended storage times of 48 to 168 h (1 week). Bars are the median value of the 2 replicates (6 injections). Experiments with the blended matrix CD were repeated in quadruplicate for the 48 h storage time and 2 times for the 1‐week storage time. The error bars are the 95% confidence interval for each quadruplicate experiment.

The reproducibility of the results was further explored by preparing 6 lots of blended matrix CD. Matrix aging (i.e., change in properties of a specific lot) was evaluated by extending the storage time of prepared matrices from 2 to 7 days. Figure 4 shows the measured diffusion coefficients. The experiments were not statistically different when compared to three 48‐h replicate experiments using reference matrix A (*p* > 0.05). After 2 weeks of storage, the matrix showed a high background fluorescence, and experiments were no longer valid based on the acceptance criteria given in Section 2.5. Therefore, the experiments must be completed within 168 h of matrix preparation in order to achieve the necessary reproducibility and satisfy acceptance criteria. However, it is recommended to complete the experiments within 48 h of matrix preparation to minimize the chance of high HA background presence.

## 4 Discussion

The ISM system could be used in conjunction with other screening assays to compare subcutaneously injected therapeutics. We postulate that diffusion is the driving force for systemic uptake of large molecules from the injection site to lymph ducts, as these large molecules (such as mAbs) are too large to pass into the venous system, so their uptake occurs through the lymph capillary network [12, 19]. Hence, measuring diffusion is important and may be correlated to protein residence time in the SQ environment and consequently contribute to in vivo bioavailability of SQ injections.

FDA and industry guidelines for the development of in vitro methods for drug discovery and drug delivery are intended to facilitate and expedite industry adoption and regulatory approval [6, 7]. For our research, these guidelines provide a framework for gauging and mitigating the effects of lot‐to‐lot changes in HA polymer size distributions on in vitro experiments and results (compare matrices A, B, and C in Tables 1 and 2). We report binary combinations that compensate for HA variability so that the resulting matrix properties and diffusion coefficients are consistent at equivalent protein (BSA and bIgG) concentrations.

The capability for generating matrices with equivalent properties from HA of different molecular weight adds to the utility of the ISM system. This minimizes introduction of additional variability in diffusion coefficients since the data are obtained on a consistent and comparable basis, while also providing insights into how different molecular weights of HA biopolymer in vivo might affect drug delivery [15]. This system also allows protein diffusion within a matrix that simulates the SQ electrostatic environment to be compared to diffusion in buffers containing added salt, or in terms of free volume diffusion theory reported in the literature [17, 20, 21].

In this work, we tested the hypothesis that matrices with comparable free volume—and consequently the same diffusion coefficients—can be prepared by combining HA of two different molecular weights by matching both the HA mass and the estimated number of HA monomers within the matrix to the reference matrix. Viscosities and diffusion coefficients equivalent to the references were achieved with blends BF and CD, confirming our hypothesis.

The correlation between rheological characteristics and polymer molecular weight is explained by the free volume (“hole” size) and particle‐to‐particle interactions within the matrix [22]. Changes in the gel volume fractions may alter protein volume fraction, particle/gel and particle/particle interactions, all of which change protein diffusion [20, 23].

While polydispersities were similar for the HA Matrix A and B, the viscosities are measurably different due to their different molecular weights (14.2 Pa.s for B vs. 7.2 Pa.s for A) (Table 2). Similar viscosities indicate equivalence of different matrices since viscosity is a function of HA M_n_ (number‐averaged molecular weight) and M_w_ (weight‐average molecular weight) (Figure 2 ). The blends that had comparable viscosities with the reference had the highest polydispersities.

Protein diffusion characteristics are also a function of salt concentration [17, 20], thus requiring that equivalent buffers be used, and the probability of vacant volumes occurring within a matrix be considered [21]. The equivalent HA blends allow the ISM to be used to compare not only different proteins, but also different buffers in which a specific protein is prepared.

The methods and criteria presented here show that equivalent matrices may be achieved within the natural variation encountered with commercially sourced HA isolated from a biological source used for matrix formulations. Hence, measurement of diffusion coefficients over a range of protein concentrations at equivalent conditions in separate but comparable HA matrices can be accomplished.

## 5 Conclusions

Protocols for formulating HA matrices in a manner that compensates for the natural variations in HA isolated from a biological source enable comparison of diffusion coefficients of a monoclonal antibody (mAb3) against b‐IgG and BSA. The developed method and analysis results in matrices whose properties are consistent and reproducible, based on HA matrices and blends whose weight‐average molecular weights range from M_W_ = 0.67 to 1.65 MDa (corresponding to M_n_ = 0.44 to 1.23) at total concentrations of 10 mg HA/mL in phosphate buffer, pH 7.4. Starting with a single lot of HA used to develop the ISM, subsequent binary combinations of different HA fractions based on characteristic viscosity of the HA and diffusion of test proteins were evaluated. This enabled us to identify matrices with similar properties suitable for consistent measurement of intra‐matrix protein diffusion. This also sets the stage for comparing diffusion coefficients of biotherapeutic proteins based on in vitro measurements, as well as to provide for broader adoption of HA‐based in vitro assays by achieving consistency across HA lots and comparability over time.

## Nomenclature

  

M_n_
: number‐averaged molecular weight
M_w_
: weight average molecular weight
MDa: mega Dalton (measure of polymer molecular weight)
pI: pH at the iso‐electric point

### Greek Symbols

  

μ: viscosity of hyaluronic acid (HA) dissolved in PBS

## Conflicts of Interest

The authors declare no conflicts of interest.

## Data Availability

The data that support the findings of this study are available from the corresponding author upon reasonable request.
